# Technostress, Quality of Work Life, and Job Performance: A Moderated Mediation Model

**DOI:** 10.3390/bs13121014

**Published:** 2023-12-15

**Authors:** Farida Saleem, Muhammad Imran Malik

**Affiliations:** 1 Department of Management, College of Business Administration, Prince Sultan University, Riyadh 11586, Saudi Arabia; 2 Department of Management Sciences, COMSATS University Islamabad, Attock Campus, Attock 43600, Pakistan; im4imranmalik@gmail.com

**Keywords:** technostress, quality of work life, organizational flexibility, job performance, transactional model of stress and coping

## Abstract

This study examines the effect of technostress on teachers’ quality of work life and job performance. A moderated mediation model is proposed and tested based on the transactional model of stress and coping. This study proposes organizational flexibility as the boundary condition—a first-level moderator—and quality of work life as the explanatory variable. A sample of 199 university teachers who worked from home or used the hybrid teaching mode was selected. Data were collected through closed-ended questionnaires. Structural equation modeling (SEM) and the Hayes PROCESS Macro (extension in SPSS) were used for hypothesis testing. The results found that the three dimensions of technostress (Techno complexity, Techno invasion, and Techno overload) negatively and significantly affect teachers’ quality of work life. However, there are significant positive direct effects of these three dimensions of technostress on employee performance and significant negative indirect effects on performance through quality of work life. Organizational flexibility acts as a significant moderator, where a low value of organizational flexibility enhances the negative relationship between technostress and quality of work life. In contrast, high values of organizational flexibility convert the significant negative relationship into an insignificant impact. The university management must take measures to overcome technostress among teachers by showing flexibility.

## 1. Introduction

The use of technology helps to streamline the processes and procedures of operations in an organization. However, it can also create ambiguity and stress for employees who are forced to use it. Organizations’ forced use of technology is linked with employee stress [1], or more precisely, technostress [2]. Technostress, like any other stress, can have negative consequences for employers; hence, more attention is required in general and in education settings in particular [2]. Teachers’ working conditions drastically changed during the pandemic, affecting their quality of life [3]. Teachers generally commit highly to their work, job satisfaction, and quality of life. However, excessive use of technology does not always result in productive outcomes. Thus, it is important to examine the factors that influence the quality of work life and performance of teaching professionals [4]. According to the Teacher Wellbeing Index 2018, two out of five teachers in the UK struggle with mental health and well-being. In less than five years, 52% of professionals left their profession. Burnout and workload led more than half of US teachers to consider quitting their jobs [5]. The situation might be worse in developing countries with limited resources to tackle the negative effects of technology.

The literature has reported mixed findings related to the impact of technostress on employee outcomes. For example, AL-Ansari and Alshare [6] found significant negative effects of technostress on job satisfaction, Tarafdar et al. [7] found a negative association between technostress creator and performance, and Saidy et al. [8] found four out of five dimensions of technostress negatively influencing both in-role and extra-role performance. However, Saleem et al. [9] found a significant positive impact of technostress on the performance levels of university teachers. Technostress in the work environment is attributed to positive and negative consequences; however, research is scarce regarding the explanatory and boundary mechanisms for technostress and employee performance. With this research, we have attempted to fill this gap in the literature by empirically investigating a holistic model based on the Transactional Model of Stress and Coping while proposing quality of work life as an explanatory factor and organizational flexibility as the boundary condition.

The work environment plays a significant role in teachers’ work lives and affects their motivation levels [10]. Unfortunately, the enforced and excessive use of technology during and after COVID-19 impacted teachers’ psychological and physical health [11]. These tools and systems have brought ease and convenience by increasing the pace of work and allowing multi-tasking, but they are also linked to creating stressful situations [9]. Adopting and using technology can be time-consuming and problematic [12]. Mental fatigue, skepticism, and ineffectiveness are some of the outcomes of technostress [13], which likely hinder teachers’ quality of work life.

Similarly, organizational flexibility can help an organization anticipate and adjust to uncertain situations [14]. Flexibility allows an organization to make significant changes without sacrificing its employees’ commitment and dedication [15]. The use of information and communication technologies also supports organizational flexibility. It has positive implications for organizational performance and the quality of employees’ work lives [16]. Flexibility options, such as offering flexible places and flexible time, can enable teachers to spend additional time and energy on themselves [16] and can be linked to positive outcomes for employers and employees.

This study contributes significantly to the existing literature in many ways. First, we proposed a moderated mediation model based on the Transactional Model of Stress and Coping [17] to study the impact of technostress experienced by university teachers and their performance while proposing quality of work life as a mediator and organizational flexibility as a moderator. Second, many predictors of teachers’ quality of work life have been examined in the prior literature [18,19,20]; however, very few have considered technostress in university teachers. Similarly, very few researchers have empirically examined this in Asian and developing countries. Third, the literature examining teachers’ quality of work life has not considered organizational flexibility as a boundary condition. Lastly, this study will help generalize theories and concepts that have largely been developed in Western and European contexts.

## 2. Literature Review

### 2.1. Transactional Model of Stress and Coping

The transactional stress and coping model developed by Lazarus and Folkman [16] explained that coping involves cognitive and behavioral responses that individuals employ when they perceive that stressors are beyond their capabilities. They found that an individual’s stress is closely tied to their confidence in handling a threat. The differing impacts of different techno-stressors may stem from individuals perceiving them differently in specific situations. Recent studies have also shown that users may assess certain techno-stressors as challenges while viewing others as threats, leading to distinct effects [21,22,23]. For example, Zhao et al. [22] discovered that individuals tend to regard techno-overload and techno-uncertainty as challenges while considering techno-complexity and techno-insecurity as threats.

Numerous prior studies have used the transactional model of stress and coping to investigate the effects of techno-stressors [21,23,24]. For instance, Lei and Ngai [24] were pioneers in adapting transactional stress and coping models to the context of technostress and investigated the conflicting findings concerning the impact of technostress. Following this, Chen et al. [25] explored how mobile shoppers assess technostress to gain insights into discontinuation behavior. Zhao et al. [22] examined the ramifications of technostress on employee productivity by analyzing appraisal and coping processes. Wang and Yao [21] examined how K-12 teachers evaluate techno-stressors. Similarly, while using the transactional stress and coping model, Wang, Zhao, and Yao [23] investigated tech-no-stressors related to online teaching during the COVID-19 pandemic, a period when teachers faced an increased risk of experiencing technostress.

The transactional stress model, as developed by Lazarus and Folkman [16], can be applied to understand and analyze technostress in the context of teachers’ quality of work life. The transactional stress model provides insights into how interventions, such as support programs and organizational flexibility, can be designed to help teachers effectively manage technostress and maintain a positive work-life balance. Hence, based on the transactional stress model and coping, it is proposed that university support in the form of providing flexibility in operations and a cooperative culture will help mitigate the negative impact of technological stress on the quality of the work-life of university teachers.

### 2.2. Technostress and Employee Performance

Penado et al. [26] emphasize that, primarily because of the ongoing changes stemming from scientific and technological advancements that have unfolded since the 1990s, teaching has become one of the most stress-inducing professions globally. The role of a teacher has transformed from being a mere knowledge transmitter to a complex creator of learning environments, where technology plays a pivotal role as a method of instruction [26]. Teachers today are tasked with managing the dynamic interplay among the three central components of the learning environment, i.e., content, pedagogy, and technology. Teachers must adeptly and effectively incorporate technology into their classroom teaching [27]. However, they need help with the limited time available to keep up with emerging technologies and innovations in pedagogy [28,29]. Teachers’ capacity to effectively integrate technology into pedagogy is vital for fostering educational innovation [30,31]. The continuous evolution of technology puts teachers under persistent technostress because they might not always possess the requisite knowledge to use these new and updated technologies [32,33].

Technostress negatively impacts an individual’s psychological and physiological well-being [34]. They become overburdened and lose concentration, which results in lower performance [8]. While studying technostress in Spanish university teachers, Penado et al. [26] concluded that with the increased use of technology in education, university teachers face technostress that affects their performance. Technostress can decrease job satisfaction, result in burnout, and decrease productivity among university teachers [35]. Using data from China, Li and Wang [36] found that technostress creators (tech-no-complexity and techno-invasion) negatively affect university teachers’ work performances. This study showed that technostress led to teachers’ inability to manage their time and workload effectively and had negative consequences for performance.

Similarly, Chapay and Bangoc II [37], while studying the relationship between technostress and performance among Filipino teachers, found a significant negative impact of technostress on work performance. As supported by literature findings, technostress is not a unidimensional construct. Techno complexity, techno invasion, and techno overload are important dimensions of technostress. Based on the literature findings and the above discussion, the following hypotheses are proposed:

**H1a.**  *Techno complexity negatively affects teachers’ performance.*

**H1b.**  *Techno invasion negatively affects teachers’ performance.*

**H1c.**  *Techno overload negatively affects teachers’ performance.*

### 2.3. Technostress and the Quality of Work-Life

Quality of work life (QWL) is the extent to which employees are satisfied with their personal needs and the work experience they obtained from an organization [38]. According to Sirgy, Efraty, Siegel, and Lee [39], the quality of work-life refers to an organizational environment in which employees feel safe and secure and can work with diligence. Various factors affect the quality of work life of employees, including employees’ welfare and health care, incentive programs, employment security, job adequacy, job design, growth, and development opportunities, reducing conflicts and ambiguities, participation in decision-making, and training and reward systems [40,41,42,43]. It has also been suggested that employees’ safety and protection from stress-producing factors, including technostress, are required to enhance their proficiency and quality of work life [44].

According to Gordani et al. [45], “Teachers’ Quality of Work Life (QWL) is a comprehensive concept that reflects a teacher’s level of contentment regarding their job and overall work circumstances”. Technostress creates uneasiness, emotional exhaustion, skepticism, and functional impairment in employees [12] and can be linked to negative effects on teachers’ quality of work life. Prior studies have also shown that technostress has a harmful impact on people’s overall quality of life [46,47]. An unfavorable working environment and excessive workload reduce teachers’ work-life quality [48]. Quality of work life is significantly positively correlated with teachers’ mental health [43], which is necessary for effective teaching. Considering the above arguments, it is posited that stress due to technology can lead to decreased teachers’ quality of work. Hence, the following hypotheses are proposed:

**H2a.**  *Techno complexity negatively affects teachers’ quality of work life.*

**H2b.**  *Techno invasion negatively affects teachers’ quality of work life.*

**H2c.**  *Techno overload negatively affects teachers’ quality of work life.*

### 2.4. Quality of Work Life as a Mediator

A positive QWL often includes factors such as manageable workloads and supportive work environments. When teachers can maintain a healthy work-life balance, they are less likely to experience burnout, and their performance remains consistent and adequate [49]. Teachers with a good QWL experience lower stress levels and better emotional well-being. This emotional stability allows them to manage the challenges of teaching more effectively and maintain a positive classroom environment [50]. QWL is an important mediator between technostress and university teachers’ performance. By improving QWL through interventions such as training and support, universities can help reduce the adverse effects of technostress and improve teachers’ performance. Based on the above discussion, the following hypothesis is proposed:

**H3a.**  *The quality of work life mediates techno complexity and teachers’ performance relationship.*

**H3b.**  *The quality of work life mediates techno invasion and teachers’ performance relationship.*

**H3c.**  *The quality of work life mediates techno overload and teachers’ performance relationship.*

### 2.5. Organizational Flexibility as a Moderator

Organizational flexibility refers to an organization’s ability to adapt to environmental and technological changes [51]. The degree of flexibility in an organization depends on its ability to anticipate and respond to uncertain situations [13,52]. Flexible organizations can respond more efficiently to environmental challenges and demands than rigid organizations. Organizations require flexibility to survive in unpredictable and changing environments [52]. Flexible organizations respond effectively to external environmental changes through the fast and efficient allocation of resources [53]. Increasing flexibility means strengthening management control by having various managerial capabilities and being able to move quickly [14]. Most employees feel less stressed and more grateful to their employers when they have flexible policies [54]. It increases work-life quality, and employees tend to be more engaged, satisfied, and have a work-life balance, leading to increased retention and commitment [55].

While there is ongoing debate regarding the advantages of flexible work arrangements for businesses and potential challenges for employers, these policies are always advantageous for employees [56]. Flexible practices are supported by the university, which makes teachers satisfied and committed. Researchers have noted that adopting family-friendly policies (e.g., flexible working) reduces conflict and improves work-life quality [56]. Flexible policies and working conditions help reduce stress levels. Because of organizational support and flexibility, employees try to perform better in reciprocity and feel satisfied with a higher quality of work life. Organizational flexibility can moderate the relationship between technostress and university teachers’ well-being, as it allows them to cope with the stress caused by technology use. Based on the above discussion, the following hypothesis is proposed:

**H4.**  *Organizational flexibility attenuates the negative relationship between technostress and teachers’ quality of work life.*

Organizational flexibility allows university teachers to adjust and use technological changes effectively. Organizational flexibility can positively impact university teachers’ performance, allowing them to manage their workload better, resulting in improved job satisfaction and increased productivity [57]. Bran and Udrea [58] state that “the flexibility itself can become a strong non-financial motivation.” While motivation is generally conducive to achieving better performance, flexibility significantly enhances performance levels. Flexibility fosters a positive attitude and enables employees to focus more effectively on their objectives [58].

This study posits that technostress is a significant source of stress for university teachers and can negatively impact their performance [59]. However, organizational flexibility can moderate the relationship between technostress and university teachers’ performance by allowing them to adapt to and use technological changes effectively. Based on the above discussion, the following hypothesis is proposed:

**H5.**  *Organizational flexibility attenuates the negative relationship between technostress and teachers’ performance.*

**Figure 1:**
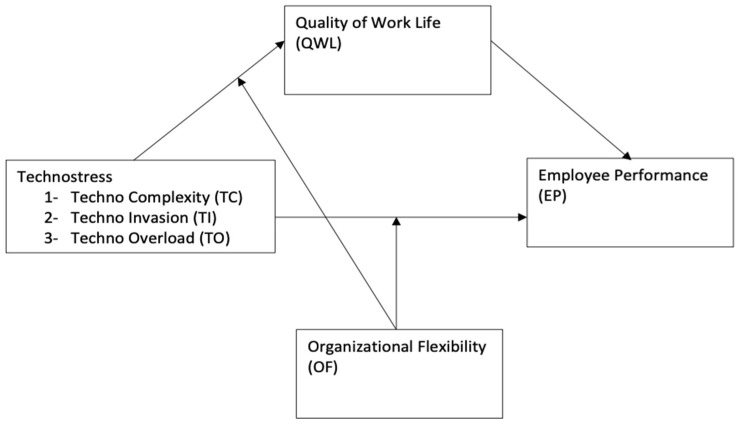
The Research Framework.

The proposed research framework based on the above literature review is presented in Figure 1.

## 3. Methodology

### 3.1. Sample and Data Collection

A Google survey form were used for data collection. The authors used their references to distribute the electronic version of the survey form to different public and private sector universities in Pakistan. The snowball sampling technique was used to generate the responses. Snowball sampling is a recruitment technique in which research participants are asked to assist researchers in identifying other potential subjects. The target respondents were university teachers only. A total of 213 responses were received. A total of 14 responses were excluded due to missing values, and 199 responses were used for the final analysis. The demographic characteristics of the sample were analyzed using SPSS. It shows that most respondents were male (61.8%). Most respondents were from the age groups of 31–40 (54.8%). Approximately 67% of respondents had higher qualifications (MS) or Ph.D. degrees, while 48% were from public sector universities. The demographic information of the respondents is shown in Table 1.

### 3.2. Instrumentation

A closed-ended survey form was used to collect responses. QWL was measured using a 16-item scale adopted from Sirgy et al. [39]. All items were measured on a 5-point Likert scale ranging from “very untrue” to “very true," where “very untrue” was “1” and “very true” was “5”. The sample item is “My job does well for my family.”

Technostress was measured with the help of a 14-item scale adopted from Tarafdar et al. [60]. The scale comprises fourteen statements with three underlying dimensions: techno-overload, techno-complexity, and techno-invasion. The sample statements are “I am forced by technology to work much faster,” “I am forced by technology to do more work than I can handle,” and “I am forced by the technology to work with very tight time schedules.” All items were measured on a 5-point Likert scale ranging from “strongly disagree” to “strongly agree," where “strongly disagree” was “1” and “strongly agree” was “5”.

The scale used to measure the extent of organizational flexibility was adapted from Koçyiğit and Akkaya [61]. The responses were measured using a 5-point Likert scale ranging from “strongly disagree” to “strongly agree," where “strongly disagree” was “1” and “strongly agree” was “5”. The sample item is “Enabling employees to take the initiative for work”.

The contextual employee performance scale by Koopmans et al. [62] was used to measure employee performance. The scale comprises seven items. The sample items are “In the past three months, I took on extra responsibilities.” and “In the past three months, I took on challenging work tasks when available.” All items were measured on a 5-point Likert scale ranging from “strongly disagree” to “strongly agree," where “strongly disagree” was “1” and “strongly agree” was “5”.

### 3.3. Data Analysis Strategy

Confirmatory factor analysis (CFA) output was used to assess reliability and validity. To identify the control variables, a one-way ANOVA test was run. A sequential approach for testing the proposed hypotheses was used. Structural Equation Modeling (SEM) was used to test the first three sets of hypotheses, and PROCESS Macro by Hayes [63] was used to test the full moderated mediation model and Hypotheses H4 and H5. PROCESS Macro was preferred because it could calculate the conditional direct and indirect effects (mediated effects in the presence of a moderator) using 95% biased corrected confidence intervals. For PROCESS Macro model analysis, summative scores for each variable were used. For technostress, the composite score for each dimension was calculated by first averaging only the items related to a given dimension. After that, a composite score for technostress is derived by averaging the scores of each dimension. We have used only composite scores for technostress as the focus of the current investigation was to see the role of quality of work life and organizational flexibility in the relationship between technostress and employee performance.

## 4. Results

### 4.1. Control Variables

To check the control variables, we used a one-way ANOVA. The results indicated that only organization type was significantly related to technostress (F = 4.34, *p* = 0.04), whereas the other demographic variables, including Age, Gender, Qualification, and Experience, were insignificant. Hence, we controlled the organization type while conducting further analysis.

### 4.2. Common Method Variance

Herman’s single-factor analysis was used to check the common method variance. Exploratory factor analysis used principal component analysis with no rotation, and all measured items were loaded into a single factor. The single factor explained about 32% of the variance below the recommended threshold of 50% [64]. Hence, there was no issue of common method variance to report.

### 4.3. Confirmatory Factor Analysis

The psychometric properties of the measures were examined through confirmatory factor analysis (CFA) based on the four-factor model, namely TS, QWL, OF, and EP. The CFA resulted in an acceptable fit (CFI = 0.94; GFI = 0.74; AGFI = 0.71; RMSEA = 0.06, χ^2^ = 1599; d.f. = 927; χ^2^/d.f. = 1.74; *p* < 0.001).

### 4.4. Reliability Analysis

Scale reliability is commonly measured using two indicators. These are Cronbach’s alpha and composite reliability. A Cronbach alpha of more than 0.70 is required to establish internal consistency in a construct. Each latent construct shown in Table 2 has a Cronbach alpha greater than 0.70, indicating high internal consistency and reliability [65]. Composite reliability (CR) has been identified by Hair et al. [66] as an effective method for determining the reliability of scales. To establish internal reliability, the composite reliability must be greater than 0.70. The greater the composite reliability value, the more reliable it is. As shown in Table 2, the composite reliability of each latent construct is over 0.90, indicating internal consistency.

### 4.5. Convergent Validity

Similarly, to establish indicator convergent validity, the loadings of each observed variable should be greater than 0.60 [66]. Indicators with loadings less than 0.70 but more than 0.5 have been retained because the average variance extracted (AVE) and composite reliability (CR) were not improved after their removal. However, two items from Organizational Flexibility (OF11, OF12) and two items from Technostress (TO4, TO5) have been dropped because of their cross-loadings. The reliability coefficients and factor loadings explaining the convergent validity are presented in Table 2.

### 4.6. Discriminant Validity

The values of AVE were higher than the cut-off value of 0.5. The discriminant validity of the collected data were checked using the criteria proposed by Fornell and Larcker [67]. According to this criterion for the assurance of discriminant validity, the AVE values of all latent variables should be greater than the shared variance of each variable. For all variables presented in the model, the AVE values were greater than the shared variances. Hence, discriminant validity was achieved (see Table 3).

### 4.7. Hypotheses Testing

After the fitting of the measurement model, the structural model was analyzed. AMOS 23 was used for SEM analysis. The maximum likelihood method, using both latent and observed variables, was utilized. The structural model was constructed using three dimensions of technostress (TC, TI, and TO) as IVs, QWL as a mediator, and EP as a DV. Table 4 shows that all three dimensions of technostress have a significant direct effect on employees’ performance (TC: β = 0.439, *p* < 0.10; TI: β = 0.189, *p* < 0.000; TO: β = 0.396, *p* < 0.000). Hence, H1a, H1b, and H1c are accepted but in opposite directions. The three dimensions have a significant but negative impact on the quality of work life (TC: β = −0.365, *p* < 0.000; TI: β = −0.119, *p* < 0.10; TO: β = −0.303, *p* < 0.000), providing support for H2a, H2b, and H2c. At the same time, quality of work life (β = 0.340, *p* < 0.000) has a significant positive impact on employee performance. The direct effect of TC, TI, and TO is positive, while the indirect effect through QWL is negative. The results support the partial mediation model, where QWL explains how the positive impact converts into a negative effect. The results for the paths examined are provided in Table 4.

**Figure 2:**
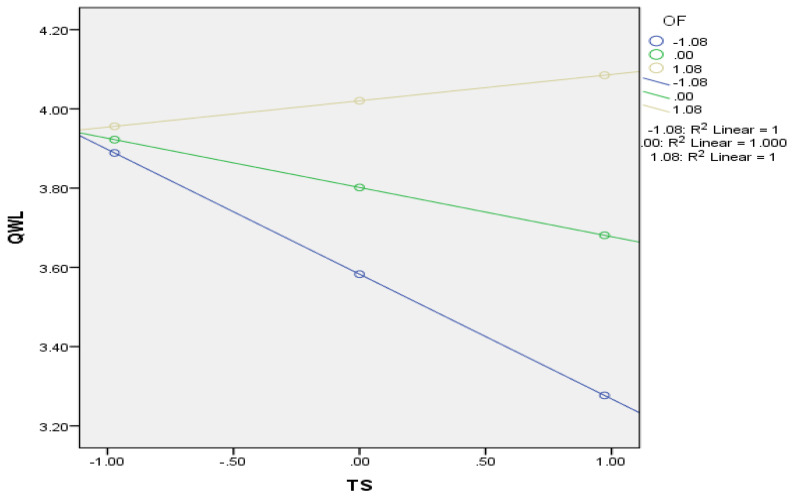
Interaction Plot of TS and OF.

After identifying that three dimensions of technostress have a similar impact on employee performance and their quality of work life, we used Process Model 8 to test our full-moderated mediation model. OF was modeled as a moderator for the effect of TS on QWL and TS on EP. This step added the OF and TS interaction to the regression equation predicting QWL, and then the OF and TS interaction to the regression equation predicting EP in the presence of QWL. In summary, TS positively and significantly impacts EP (β = 0.203, t (199) = 2.79, *p* < 0.006, *sr*^2^ = 0.10). The impact is significant but positive and not negative, as proposed. TS has a significant negative impact on QWL (β = −0.12, t (199) = −2.00, *p* = 0.046, *sr*^2^ = 0.10) and a positive impact on EP (β = 0.149, t (199) = 3.08, *p* < 0.002, *sr*^2^ = 0.71). OF significantly moderated the relationship between TS and QWL (β = 0.176, t (199) = −0.315, *p* < 0.001, *sr*^2^ = 0.10), such that at the lower values of OF, the negative relationship between TS and QWL is significant, whereas it becomes insignificant at higher values of OF. The effect size of TS on QWL at a low OF value is negative, stronger, and significant (−0.135; *p* < 0.001), and at a high OF value, it is low and insignificant (0.066; *p* > 0.10). This means that the negative relationship between technostress and quality of work life is stronger when there is less flexibility in the organization, while this negative impact becomes insignificant when the organization is flexible, leading to the acceptance of H4. The harmful impact of technostress on the quality of work life is notably aggravated in environments characterized by limited organizational flexibility. In such settings, the negative relationship between technostress and the quality of work life becomes more pronounced and substantial. Conversely, when organizations demonstrate a higher degree of flexibility, this negative association diminishes to the point of insignificance. This highlights the intensifying influence of technostress in less flexible organizational structures and underscores the crucial role of organizational flexibility in mitigating the adverse effects on employees’ work-life quality. The interaction plot explaining the relationship between technostress and QWL at different values of organizational flexibility is presented in Figure 2. The interaction of TS and OF in predicting EP in the presence of QWL is insignificant, leading to the rejection of H5. The results of Process Model 8 are presented in Table 5.

In addition to the hypothesized relationships, we tested the conditional indirect effect in the presence of a first-stage moderator. Table 5 shows that the magnitude of the indirect effect of TS on EP through QWL (−0.0468) is strongest and negative when OF is low. The results of the moderated mediation analysis show that the conditional direct effect of TS at different values of OF is insignificant. In contrast, the conditional indirect effects of TS on EP through QWL in the presence of OF are significantly negative at low values of OF and become insignificant at high values of OF. The index of moderated mediation is also significant, and there is no zero in the CI (Index: −0.0261; CI: 0.037; 0.065), indicating that the proposed model is a moderated mediation model.

## 5. Discussion

With this study, we have assessed the impact of technostress on teachers’ performance in university settings. The quality of work life was examined as a mediator, and organizational support in the form of organizational flexibility was examined as a moderator. The first set of hypotheses looking at the impact of three dimensions of technostress (techno complexity, techno invasion, and techno overload) on university teachers’ performance are accepted, but in the reverse direction. The results show that all three dimensions of technostress (TC, TI, and TO) have a significant but positive relationship (Hypotheses H1a–H1c). Technostress is not negatively affecting teachers’ performance. According to the literature, university teachers are particularly susceptible to technostress because of their heavy reliance on technology [68]. The significant and positive effect of technostress on teachers’ performance is in line with the findings of Li and Wang [33], who have identified techno overload as positively associated with the work performance of university teachers. However, Penado et al. [26] found that technostress was not a predictor of the performance of Spanish university teachers during COVID-19.

Similarly, Saleem et al. [9] also found that university teachers perform well despite technostress during COVID-19. Our findings suggest that technostress does not necessarily lead to a decline in performance among university teachers. Since technostress was not negatively associated with performance, it is plausible that the emergency situation generated by COVID-19 has pushed universities to adapt technologies. As the data were collected in mid-2022, teachers had time to align themselves with the required technological skills and appreciate the positive side of working with technologies.

The second set of hypotheses, which examined the effect of techno complexity, techno invasion, and techno overload on university teachers’ quality of work life (Hypotheses H2a–H2c), are accepted. All three dimensions of technostress (TC, TI, and TO) significantly negatively impact the quality of work life. The use of technology has a significant impact on individuals’ work-related stress, anxiety, and dissatisfaction [69]. In education, teachers are increasingly required to use technology to enhance their teaching and communication with students, but this can also result in a decreased quality of work life [35].

Technostress can affect teachers’ quality of work life through increased workload and time pressures. Teachers must manage multiple digital platforms, respond to emails and messages from students and parents, and keep up with the latest technology trends, which can lead to overwhelming workloads and burnout. This, in turn, can lead to decreased job satisfaction, emotional exhaustion, and a lower commitment to teaching [40,41]. Another way technostress affects teachers’ quality of work life is through increased interruptions and distractions. Teachers may have to deal with technical glitches, slow internet speeds, and a barrage of notifications, which can disrupt their concentration [7]. To mitigate the negative effects of technostress on teachers’ quality of work life, educational institutions can provide training and support to help teachers use technology effectively, efficiently, and confidently [9]. Institutions can also promote the adoption of policies and practices that encourage a healthy work-life balance, such as flexible schedules, reduced workloads, and opportunities for professional development [7]. The third set of hypotheses regarding the mediating role of quality of work life is also partially accepted. The results also show that quality of work life partially mediates the relationship between techno complexity, techno invasion, and techno overload and teachers’ job performance. The relationship between technostress, QWL, and job performance is complex and dynamic. The results show that techno complexity, techno invasion, and techno overload affect teachers’ QWL, which, in turn, affects their job performance.

Hypothesis H4, organizational flexibility moderates the relationship between technostress and teachers’ quality of work life, was also accepted. The ability of an organization to adapt and change in response to internal and external factors can have positive implications for employee performance. The conditional effect of technostress on teachers’ quality of work life was significantly negative at lower levels of organizational flexibility and was non-significant at high levels of organizational flexibility. Our results suggest that the negative impact of technostress on teachers’ quality of work life may depend on the level of organizational flexibility within their educational institutions. This finding is supported by Bran and Udrea [58], who found support for a positive impact of flexibility on job performance. In universities, organizational flexibility can involve the implementation of policies and practices that support teacher autonomy and decision-making [56].

Similarly, our study also found that the negative effect of technostress on the quality of work life was significantly stronger at lower levels of organizational flexibility. When there is no flexibility or a lower level of flexibility in the organization, the technostress adversely affects the quality of work life. When organizational flexibility is high, the adverse effect of technostress becomes insignificant. Increasing organizational flexibility can help reduce technostress’s negative impact on teachers’ quality of work life. This highlights the importance of creating supportive work environments that encourage teacher autonomy and decision-making power in the context of technology use.

These findings highlight the importance of organizational flexibility in supporting teachers’ well-being when using technology. By providing teachers with more autonomy and decision-making power, universities may be able to mitigate the negative effects of technostress on their quality of work life. Therefore, university administrators should create a flexible, supportive work environment that values teacher input and encourages collaboration and innovation [56,58].

The hypothesis that organizational flexibility moderates the relationship between technostress and teachers’ performance was not supported. However, the conditional indirect effects of technostress on the quality of work life are negative and significant at lower levels of organizational flexibility. These negative indirect effects become insignificant at high levels of OF. The various organizational factors may influence the relationship between technostress and job performance, and organizational flexibility may play a role under certain conditions. The direct effects of technostress on teachers’ job performance in the presence of quality of work life and organizational flexibility are insignificant. Similarly, the conditional indirect effects of technostress on job performance are moderated by organizational flexibility. Therefore, organizations need to consider not only the level of technostress experienced by employees but also the level of organizational flexibility when attempting to improve job performance and reduce the negative effects of technostress.

## 6. Conclusions

In conclusion, this research study provides valuable insights into the impact of technostress on university teachers’ performance and quality of work life. The findings reveal that technostress (techno complexity, techno invasion, and techno overload) has a significant and positive effect on teachers’ performance but a negative effect on their quality of work life. Furthermore, the quality of work life acts as a mediator between technostress and the teacher’s performance. This study also found that organizational flexibility moderates the relationship between technostress and teachers’ quality of work life but not their performance. However, the conditional indirect effects of organizational flexibility on both relationships are significant, indicating that lower levels of organizational flexibility lead to negative indirect effects.

### 6.1. Theoretical and Practical Implications

The transactional stress model [17] provided an understanding and analysis of technostress in the context of teachers’ quality of work life. The transactional stress model recognizes that stressors and coping strategies can evolve over time. As teachers gain more experience with technology and receive additional support, their appraisals and coping mechanisms may change. This creates a feedback loop where ongoing assessment and adaptation are crucial for maintaining teachers’ quality of work life despite technostress. The transactional stress model explains how teachers appraise technological challenges, coping strategies, and resulting outcomes. This framework provides insights into how interventions, such as support programs and organizational flexibility, can be designed to help teachers effectively manage technostress and maintain a positive work-life balance.

The results of our investigation identified the positive and significant impact of technostress on performance. The data for the current investigation were collected during Pakistan’s economic recession. Many people have lost their jobs during COVID-19 and due to the economic conditions in Pakistan. The threat of losing jobs and economic conditions can be attributed to the positive impact of technostress on performance. Franke and Kaul [70] also found that the threat of losing a job in an economic depression may partially explain the increase in productivity. Similarly, Wickström and Bendix [71] have also concluded that the increase in productivity in adverse conditions can be an outcome of “(i) relief from harsh supervision, (ii) receiving positive attention, (iii) learning new ways of interaction, (iv) possibilities to influence work procedures, (v) rest pauses, (vi) higher income, or (vii) the threat of losing one’s job.” [71], p. 366.

The practical implications are wider than those of university administrators. Policymakers should recognize the harmful negative effects of technostress on teachers’ quality of work life and take proactive measures to mitigate these impacts. For example, implementing flexible policies and offering training programs to promote technological literacy among teachers could significantly reduce technostress and improve their quality of work life. The findings also highlight the importance of prioritizing the quality of work life of university teachers to enhance their performance, as their productivity is critical to the success of educational institutions.

### 6.2. Limitations and Future Directions

This study has a few limitations to report. First, this study is based on a cross-sectional design, which may limit the ability to make causal inferences. A longitudinal study design would allow for examining changes in technostress, teacher’s performance, quality of work life, and organizational flexibility over time, which could provide more conclusive evidence of these variables’ mediating and moderating effects on job satisfaction and turnover intentions. Second, this study relies on self-reported data, which may introduce social desirability bias and may not reflect actual behaviors or perceptions. Similarly, the sample population demographics might also have an impact on the findings and can raise issues related to the generalizability of the results to a broader population. Future studies could use self-reported data and objective measures, such as performance metrics or organizational records, to better understand the relationships between technostress, quality of work life, and performance.

Third, because this study is context-specific, future investigations can study the proposed model in a different organizational setting. This would allow for examining potential differences in the relationships among technostress, quality of work life, organizational flexibility, and job performance across different groups. Fourth, this study only examines the mediating and moderating effects of quality of work life and organizational flexibility. Future studies could investigate other variables that may impact technostress, teachers’ quality of work life, and job performance, such as job demands, social support, job autonomy, employee engagement, and organizational commitment. Finally, this study could also be extended by examining the potential impact of interventions to improve the quality of work life and organizational flexibility on job satisfaction and turnover intentions.

## Figures and Tables

**Table 1:** Demographic Analysis.

Variables	Categories	Frequency	Percent
Gender	Male	123	61.8%
	Female	76	38.2%
Age (years)	20–30	61	30.7%
	31–40	109	54.8%
	41–50	24	12.1%
	50 and above	5	2.5%
Qualification	Masters (16 years)	66	32.7%
	MS/Ph.D. (18 years)	133	67.3%
University	Public	95	47.7%
	Private	104	52.3%
Experience (years)	1–10	126	63.3%
	11–15	36	18.1%
	16 and above	37	18.6%

**Table 2:** Results of Confirmatory Factor Analysis (CFA).

Construct/Variable	β	Alpha	CR	AVE
Techno complexity		0.950	0.951	0.665
TC1	0.812			
TC2	0.989			
TC3	0.813			
TC4	0.719			
TC5	0.638			
Techno Overload				
TO1	0.823			
TO2	0.968			
TO3	0.816			
Techno Invasion				
TI1	0.862			
TI2	0.918			
TI3	0.936			
TI4	0.924			
Quality of Work Life (QWL)		0.940	0.939	0.673
QWL1	0.748			
QWL2	0.845			
QWL3	0.852			
QWL4	0.817			
QWL5	0.866			
QWL6	0.826			
QWL7	0.826			
QWL8	0.773			
QWL9	0.761			
QWL10	0.793			
QWL11	0.820			
QWL12	0.853			
QWL13	0.837			
QWL14	0.854			
QWL15	0.825			
QWL16	0.775			
Organizational Flexibility (OF)		0.969	0.971	0.769
OF1	0.830			
OF2	0.865			
OF3	0.886			
OF4	0.888			
OF5	0.905			
OF6	0.896			
OF7	0.877			
OF8	0.884			
OF9	0.869			
OF10	0.868			
Employee Performance (EP)		0.940	0.949	0.729
EP1	0.736			
EP2	0.820			
EP3	0.881			
EP4	0.866			
EP5	0.880			
EP6	0.898			
EP7	0.850			
Goodness of Fit Indices				
χ^2^ = 1599; d.f. = 927; χ^2^/d.f. = 1.74; *p* < 0.001; CFI = 0.94; GFI = 0.74; AGFI = 0.71; RMSEA = 0.06				

β: standardized coefficient; Alpha: Cronbath’s Alpha; CR: Composite Reliability; AVE: Average Variance Extracted.

**Table 3:** Descriptive statistics and correlations.

	Variable	No. of Items	Mean	s.d.	TS	QWL	OF	EP
1	TS	13	3.19	0.94	0.665			
2	QWL	7	3.83	0.87	−0.117 (0.013)	0.673		
3	OF	6	3.41	1.08	0.206 ** (0.042)	0.168 * (0.028)	0.769	
4	EP	4	3.12	1.03	0.174 * (0.030)	0.256 ** (0.065)	0.648 ** (0.420)	0.729

* correlation significant at 0.05; ** correlation significant at 0.01; Shared Variance is in parenthesis; AVE is on the diagonal.

**Table 4:** Results for Direct and Indirect Effects (Structural Equation Model).

Path	Estimate	SE	CR	*p*-Value
TC→EP	0.439	0.13	5.765	0.000
TI→EP	0.189	0.06	2.889	0.000
TO→EP	0.396	0.11	5.404	0.004
TC→QWL	−0.365	0.09	−4.422	0.000
TI→QWL	−0.119	0.05	−1.837	0.066
TO→QWL	−0.303	0.11	−5.249	0.000
QWL→EP	0.304	0.08	4.408	0.000
Standardized Effects of Technostress on Employee Performance				
Effect	Total Effect	Direct Effect		Indirect Effect
TC	0.315	0.439		−0.041
TI	0.148	0.189		−0.103
TO	0.293	0.396		−0.124
Goodness of Fit Indices				
χ^2^ = 1524; d.f. = 547; χ^2^/d.f. = 2.788; *p* < 0.001; CFI = 0.89; GFI = 0.73; TLI = 0.87 RMSEA = 0.09				

TC: Techno Complexity; TI: Techno Invasion; TO: Techno Overload; QWL: Quality of Work Life; EP: Employee Performance.

**Table 5:** 5000 Bootstrap Results for Conditional Direct and Conditional Indirect Effects. PROCESS Model 8.

Effects Using 5000 Bootstrap 95% CI				
Path (Outcome QWL)	Estimate	SE	LL 95% CI	UL 95% CI
TS	−0.124 **	0.06	−0.247	−0.002
OF	0.219 *	0.06	0.089	0.314
TS-X-OF	0.176 *	0.05	0.066	0.256
Conditional Effects of OF using 5000 Bootstrap 95% CI TS→QWL				
values of OF	Effect	SE	LL 95% CI	UL 95% CI
−1.084	−0.315 *	0.08	−0.475	−0.154
0.000	−0.124 **	0.06	−0.246	−0.002
1.084	0.066	0.09	−0.114	0.247
Conditional Effects using 5000 Bootstrap 95% CI				
Path (Outcome EP)	Estimate	SE	LL 95% CI	UL 95% CI
TS	0.018	0.04	−0.065	0.101
QWL	0.149 *	0.05	0.054	0.244
OF	0.774 *	0.04	0.697	0.852
TS-X-OF	−0.012	0.04	−0.087	0.064
Conditional Indirect Effects of OF using 5000 Bootstrap 95% CI TS→QWL→EP				
Indirect Effect at different values of OF	Effect	SE	LL 95% CI	UL 95% CI
−1.084	−0.047 *	0.02	−0.101	−0.012
0.000	−0.018	0.01	−0.042	0.001
1.084	0.009	0.02	−0.016	0.054

* *p* < 0.01; ** *p* < 0.05

## Data Availability

The data will be available upon request from the corresponding author.
